# Decolonising tertiary psychology student support in Australia: empowering Aboriginal and Torres Strait Islander psychology students

**DOI:** 10.1080/00049530.2025.2478083

**Published:** 2025-03-24

**Authors:** Belle Selkirk, Pat Dudgeon, Chontel Gibson, Joanna Alexi

**Affiliations:** a Australian Indigenous Psychology Education Project, School of Indigenous Studies, University of Western Australia, Crawley, Australia; b Indigenous Futures Centre (NT/WA Node), University of Western Australia, Crawley, Australia

**Keywords:** Decolonising psychology, indigenous psychology, student support, Aboriginal and Torres Strait Islander peoples, cultural safety, social and emotional wellbeing

## Abstract

**Objective:**

Aboriginal and Torres Strait Islander people, including psychologists, are actively leading and decolonising psychology. The focus of decolonising psychology is on epistemic justice for Indigenous knowledges and delivering culturally responsive services. Indigenous psychologists play a vital role in the decolonising process. Despite recommendations and initiatives aimed at decolonising psychology tertiary programs, such as increasing the representation of Indigenous peoples’ completing psychology tertiary education programs, completion rates remain below parity. This study explores the current strategies and initiatives within Australian psychology tertiary programs, which support Aboriginal and Torres Strait Islander psychology students.

**Method:**

Aboriginal Participatory Action Research was foundational in this qualitative research, which included an online survey. Eighteen representatives from Australian psychology higher education providers participated in an online survey. Data were analysed using qualitative content analysis.

**Results:**

Four key themes emerged: policies and structural support, partnerships with Indigenous communities, research and placement support and processes supporting cultural safety.

**Conclusions:**

Findings emphasise the need for multipronged and layered initiatives to support Aboriginal and Torres Strait Islander psychology students. Implementing these strategies can enhance the recruitment, retention and graduation of Aboriginal and Torres Strait Islander psychology students, contributing to a more culturally responsive psychology workforce.

## KEY POINTS

**What is already known about this topic:**

1. Increasing Indigenous representation in psychology is a significant component to culturally safe care with Aboriginal and Torres Strait Islander peoples.
2. One systemic approach is increasing the number of Aboriginal and Torres Strait Islander peoples in psychology education and workforce; however, the proportion of Aboriginal and Torres Strait Islander students is below parity across all higher education levels in psychology.
3. Previous research has indicated that multilayered approaches in higher education is needed to support Aboriginal and Torres Strait Islander students, but there is little information in the literature specific to psychology education.

**What this topic adds:**

1. Multipronged strategies and layered support specific to psychology education is required to effectively support Aboriginal and Torres Strait Islander students in their psychology education.
2. Four themes of student support were revealed: policies and structural support; partnerships with Aboriginal and Torres Strait Islander peoples, organisations and departments; research and placement support; and processes supporting cultural safety.
3. Future research could engage Aboriginal and Torres Strait Islander students in a comprehensive exploration of psychology-specific barriers, social and emotional wellbeing factors and lived experiences pertaining to psychology education.

The collective efforts of Indigenous leaders in psychology have paved the way in decolonising Australian psychology, including prioritising increased Aboriginal and Torres Strait Islander peoples’ representation in the profession (Clark & Hirvonen, 2022; Dudgeon et al., 2014; Selkirk et al., in press). Subsequentially, empowering Aboriginal and Torres Strait Islander students to undertake and complete higher education studies in psychology is vital to strengthening the psychology workforce. However, psychology as a discipline is entrenched in Western ideologies that continues to perpetuate colonial oppression of Indigenous knowledges (Dudgeon & Walker, 2015; Fish et al., 2024; Waitoki et al., 2018). Colonial oppression negatively impacts on wellbeing inequities, simultaneously discouraging some Aboriginal and Torres Strait Islander students from studying psychology (Cameron & Robinson, 2014; Dudgeon et al., 2016; Harris et al., 2012). Compounding the wellbeing inequities are systemic level barriers, such as institutional racism, lack of student support and other contextual factors, including socioeconomic disparities and competing obligations that contribute to the inequities faced by many Aboriginal and Torres Strait Islander students in higher education (Fredericks et al., 2022, 2023; Taylor et al., 2019).

In the psychology workforce, this inequity is seen in the substantial parity gap between Aboriginal and Torres Strait Islander psychologists and non-Indigenous psychologists. Despite psychologists representing the largest mental health workforce in Australia (Australian Psychological Society, 2019), where there are currently over 46,000 registered psychologists, less than 1% of psychologists are Aboriginal and/or Torres Strait Islander (Australian Health Practitioner Regulation Agency, 2023). That proportion is substantially below population parity of approximately 3% (Australian Bureau of Statistics, 2022). In addition, the proportion of Aboriginal and Torres Strait Islander students is considerably below parity across all higher education levels in psychology. For example, in 2021, Aboriginal and Torres Strait Islander students represented 1.8% of undergraduate psychology students, with the disparity widening at the postgraduate levels, where Aboriginal and Torres Strait Islander peoples represented 0.97% of students undertaking postgraduate coursework and 1.35% of students in research degrees (Australian Government Department of Education Skills and Employment, 2021). Thus, the current enrolment rates will not contribute to increasing Aboriginal and Torres Strait Islander peoples’ representation in psychology.

Increasing the representation of Aboriginal and Torres Strait Islander peoples in psychology is a significant component of a systemic approach to facilitating leadership of culturally safe care with Aboriginal and Torres Strait Islander peoples (Dudgeon et al., 2021; Westerman Jilya Institute for Indigenous Mental Health, n.d.). The psychology profession, in Australia and elsewhere, has recognised its contribution to colonisation and its ongoing coloniality, which continues to negatively impact on Aboriginal and Torres Strait Islander peoples’ mental health and wellbeing (Carey et al., 2017). Decolonising approaches are integral in addressing the ongoing forces of coloniality. One key approach is increasing the number of Aboriginal and Torres Strait Islander people in psychology programs and workforce. Decolonising psychology curriculum and increasing Indigenous knowledges in psychology curriculum is another approach (see Selkirk et al., 2024). Decolonising initiatives such as these are on the incline in psychology, all of which contribute to systemic changes that better support Aboriginal and Torres Strait Islander wellbeing (Clark & Hirvonen, 2022; Edwige et al., 2022; Selkirk et al., in press; Westerman Jilya Institute for Indigenous Mental Health, n.d.).

Indigenous leaders are collaborating and advocating for increased student representation in psychology via national initiatives, including the Australian Indigenous Psychology Education Project (AIPEP) and Australian Indigenous Psychologists Association (AIPA) (Clark & Hirvonen, 2022; Dudgeon et al., 2021). Instrumental in this advocacy was AIPEP’s student-focused framework, which provides guidance to higher education providers (HEPs) regarding the recruitment, retention and graduation of Aboriginal and Torres Strait Islander psychology students (Dudgeon et al., 2016). The AIPEP *Guidelines for Recruitment, Retention and Graduation of Aboriginal and Torres Strait Islander Psychology Students* (Dudgeon et al., 2016) recommends multi-layered strategies to support Aboriginal and Torres Strait Islander psychology students. In particular, the guidelines focus on the importance of: leadership and prioritisation of student support; targeted recruitment supports such as specific places in programs and scholarships; partnerships with Indigenous communities, organisations and Indigenous Education Centres (IEC); vocational pathways; embedding cultural safety across all levels of the HEP, including in their physical environment, staff body, recruitment processes and curricula; and finally, a focus on the sustainability of student support initiatives (Dudgeon et al., 2016). The AIPEP framework clearly explains that Aboriginal and Torres Strait Islander student support needs to be diverse, multi-layered and place-based; spanning across individual, school and HEP levels (Dudgeon et al., 2016). Importantly, Aboriginal and Torres Strait Islander student support extends beyond individualised support (e.g., tutoring, counselling, mentoring), to addressing systemic barriers, policy change, improving equity and increasing meaningful partnerships (Dudgeon et al., 2016).

The AIPEP *Guidelines for Recruitment, Retention and Graduation of Aboriginal and Torres Strait Islander Psychology Students* (Dudgeon et al., 2016) aligns with other strategic directions in higher education (Department of Education, 2024; Fredericks et al., 2022; Universities Australia, 2022). These strategic directions highlight the need for multilayered approaches that embed systemic changes, such as curricula reform and addressing cultural safety at universities. Department-level enablers include initiatives like partnerships with the IEC and student-level supports, including tutoring, were also considered integral to aiding Aboriginal and Torres Strait Islander university completions (Department of Education, 2024; Dudgeon et al., 2016; Fredericks et al., 2022; Universities Australia, 2022). These strategic directions are broad and yet to result in extensive research relating to student support in psychology.

Research in Aboriginal and Torres Strait Islander student support have been examined for over 30 years; however, comparatively to other fields, there has been little focus on psychology (Ohan, McMullen et al., 2023; Taylor et al., 2019). AIPEP’s *Guidance on Equity Pathways* addresses one identified gap in relation to the development of equity pathways for Aboriginal and Torres Strait Islander applicants into honours and postgraduate psychology programs (Ohan, McMullen et al., 2023). The report acknowledged attention on psychology recruitment is required, due to the nuances in pathways to psychology registration with the Australian Health Practitioner Regulation Agency (Ahpra), which disproportionately has a negative effect on the enrolment of Aboriginal and Torres Strait Islander students (Ohan, McMullen et al., 2023).

Additionally, within the past 5 years, further systematic changes that support student recruitment have occurred, including accreditation changes in psychology programs that support cultural responsiveness and forthcoming policy shifts enforcing cultural safety for registered psychologists (Australian Psychology Accreditation Council, 2019; Psychology Board of Australia, 2023a, 2023b, 2024a). These system changes align with the increased focused on growing the Aboriginal and Torres Strait Islander psychology workforce from both, an equity perspective; supporting calls to decolonise the discipline of psychology, and to meet identified needs across Aboriginal and Torres Strait Islander communities (Edwige et al., 2022).

## Rationale and aims of research

Considering recent system changes and an identified gap in the literature regarding Aboriginal and Torres Strait Islander student support in psychology, it is imperative to understand the current strategies, initiatives and actions that psychology HEPs are undertaking. Capturing the current status, both innovations and gaps, in Aboriginal and Torres Strait Islander psychology student support is important to develop strategic guidance to effectively support Aboriginal and Torres Strait Islander student experiences and success in psychology education. With this in mind, the present research aimed to fill this gap by investigating the current strategies, initiatives and actions undertaken by psychology HEPs to support Aboriginal and Torres Strait Islander psychology students. This study focused on all levels of psychology study, from undergraduate to honours, and postgraduate study, including nuanced areas such as research and placement contexts.

## Methods

### Aboriginal participatory action research

This research was undertaken in line with the methodological principles of and Indigenous Research Paradigm, called Aboriginal Participatory Action Research (APAR; Dudgeon et al., 2020). APAR is grounded in Indigenous ways of knowing, being and doing, and as such encompasses leading Indigenous scholars’ work, like Moreton-Robinson’s (2013) Indigenous Standpoint Theory, Smith’s decolonising research methodologies (Smith, 2021), and Bessarab and Ng’andu’s (2010) yarning as a research method. Given the focus of the study in Aboriginal and Torres Strait Islander psychology student support, APAR provided an appropriate and culturally-grounded framework to honour decolonising practice and Indigenous ways of knowing, being and doing. Central to APAR is a commitment to Aboriginal and Torres Strait Islander leadership, governance, co-design with stakeholders and participatory action with Aboriginal and Torres Strait Islander peoples, all of whom constructed, led and shaped the research from conception to dissemination of findings (Dudgeon et al., 2020). A short summary of the APAR elements embedded in this work are described below. Selkirk et al. (2024) provide a comprehensive illustration of the elements undertaken across the broad Scoping Study survey, from this study occurred within.

#### Element 1: Aboriginal and Torres Strait Islander research leadership

Aboriginal and Torres Strait Islander leadership is central to APAR (Dudgeon et al., 2020). Aligned with this principle, this research was led by Bardi woman, professor and leader in the profession of Indigenous psychology (PD) alongside Noongar woman, early career researcher and experienced clinical psychologist (BS) and Gamilaraay woman, experienced researcher and occupational therapist (CG). A non-Indigenous Cypriot early career post-doctoral researcher (JA) worked within this leadership framework and in alignment with the core values of the team.

#### Element 2: Aboriginal and Torres Strait Islander Governance

This research was conducted with the support of an Indigenous Expert Advisory Committee, formed to guide the Scoping Study research. Representation in this committee included the AIPEP research team, and representatives from Aboriginal and Torres Strait Islander community partners, including the Australian Indigenous Psychologists Association (AIPA), National Aboriginal Community Controlled Health Organisation (NACCHO), Australian Indigenous Allied Health Australia (IAHA) and Gayaa Dhuwi Proud Spirit Australia (GDPSA).

#### Element 3: Partnership and collaboration with key stakeholders

Stakeholder engagement was an integral focus of the research, which honoured the value of collaboration and partnership in decolonising psychology. In particular, this research involved partnership with key regulatory psychology bodies and a national Community of Practice. The AIPEP Community of Practice includes educators and academics of HEPs, from a diverse range of leadership levels (see Selkirk et al., in press). Relationship and reciprocity of the partnerships supported the implementation of, and participation in, the research, as well as co-learning with stakeholders.

#### Element 4: Implementing culturally safe and human rights-based ethics protocols

The research received ethics approval from the AIATSIS Research Ethics Committee (Reference: EO273–20210720) and acknowledgement from the University of Western Australia (UWA; Reference: 2021/ET001137). This research was guided by principles of the Australian Institute of Aboriginal and Torres Strait Islander Studies (AIATSIS) *Code of Ethics for Aboriginal and Torres Strait Islander Research* (Australian Institute of Aboriginal and Torres Strait Islander Studies, 2021) and the National Health and Medical Research Council (NHMRC) Guidelines (National Health and Medical Research Council, 2018a, 2018b). Implementation of these principles are illustrated in Elements 1, 2 and 3. Additionally, we honoured intellectual and cultural copyrights by negotiating issues like authorship inclusion and acknowledgements, prior to the commencement of research and at various points within the project. A comprehensive outline of these elements that honour intellectual and cultural property is further illustrated in Dudgeon et al. (2024). We undertook a strengths-based approach to this research, which was underpinned by the key dimensions of Gibson et al. (2020) strengths-based model and philosophical approach. For example, we analysed the broader systemic constraints and key strategies to address practices that have resulted from ongoing colonial harm.

## Materials and procedure

Aboriginal and Torres Strait Islander leadership, governance, co-design with stakeholders and participatory action with Aboriginal and Torres Strait Islander peoples underpinned the development of materials and were central in all procedures relating to this research study.

### Survey

The research was conducted through an online survey, on the Qualtrics platform. As described in Selkirk et al. (2024), the overall Scoping Study survey comprised a total of 30 questions with a focus on culturally responsive curriculum and supporting Aboriginal and Torres Strait Islander psychology students. The present study focused on the second subset of survey questions (questions 18 to 24) relating to Aboriginal and Torres Strait Islander psychology student support. In this study, the term *student support* refers to a strategy, initiative or action that facilitates the recruitment, retention and/or graduation of Aboriginal and Torres Strait Islander students in psychology education. For further detail regarding the survey development, questions and dissemination process, see Selkirk et al. (2024).

### Participants

As described in Selkirk et al. (2024), p. 18 representatives of psychology HEPs (hereon referred to as HEPs) completed the online survey. At the time of the survey, there were 42 hEPs offering APAC-accredited psychology programs, and 41 registered members with the Heads of Departments and Schools of Psychology Association (HODSPA). Representatives who completed the survey were educators and academics in HEPs from across Australia; seven of the eight Australian states and territories were represented in the survey. The eligibility criteria to participate included HEPs that offered a psychology program and required a single representative complete the survey on behalf of their HEP. Participation was voluntary and informed written consent was provided.

### Data analysis

Consistent with the approach outlined in Selkirk et al. (2024), data were analysed utilising the Schreier (2014) qualitative content analysis method and an APAR methodological approach (Dudgeon et al., 2020). Both Aboriginal researcher (BS) and non-Indigenous researcher (JA) familiarised themselves with the survey data and then developed preliminary codes. Preliminary codes were validated by Aboriginal researchers, BS and CG. Guided by the coding frame, survey data were then formally coded and grouped into themes using NVivo software (Release 1.3). Double coding was conducted by Aboriginal researcher (BS), with any queries of coding reviewed and resolved. Themes were then formed and verified by Indigenous governance (PD), including providing opportunity for feedback from the Indigenous Expert Advisory group. Credibility, dependability, transferability and confirmability of the qualitative research in this study were confirmed in the same way as described in Selkirk et al. (2024).

## Results

Four key themes reflect the central findings from the survey about supporting Aboriginal and Torres Strait Islander psychology students. These core themes were: (1) Policies and Structural Support, (2) Partnership with Aboriginal and Torres Strait Islander Peoples, Organisations and Departments, (3) Research and Placement Support, (4) Processes Supporting Cultural Safety, (5) University-wide Initiatives and initiatives that were (6) Under Development. These themes are discussed below and also displayed in Table S1 (Supplementary Material).

### Theme 1: Policies and structural support

A system-based approach, which applies policies, procedures and structural supports, is integral for supporting Aboriginal and Torres Strait Islander students. Participants identified a range of policy and structural supports, which were embedded across multiple levels within HEPs. These supports encouraged recruitment, retention and graduation of Aboriginal and Torres Strait Islander students, in both undergraduate and postgraduate programs.

Indigenous equity pathways were commonly reported, and they included both pre-admission and admission policies. A key pre-admission strategy for Indigenous equity pathways, in both honours and postgraduate programs, was recruitment outreach. Key strategies in admission policies included a minimum threshold criterion for entry into honours studies and reserved places in psychology honours and postgraduate programs. One participant reported:

We have an alternative entry pathway into Honours – there are a specified number of places held for Aboriginal and/or Torres Strait Islander applicants, provided minimum entry criteria are met, in accordance with minimum entry criteria for Honours set by the University. (Participant 9)

Recruitment outreach was another identified strategy to increase Aboriginal and Torres Strait Islander representation in undergraduate and postgraduate studies. In addition, some participants reported that personalised processes were in place to identify and support admission of Aboriginal and Torres Strait Islander students to psychology programs:

The Masters program has an admission pathways policy that identifies Indigenous students whose applications will then be reviewed personally by the Head of Discipline to allow for individualised assessment of their application and accommodation regarding their previous academic performance for entry into the program. (Participant 3)

Structural supports for Aboriginal and Torres Strait Islander students in psychology programs included scholarships, mentoring programs and personalised attention and catch ups, aimed at supporting students throughout their psychology degrees. While the survey specifically asked about the unique initiatives led by the psychology HEP, a common thread was the broader role of universities in providing centralised student support. Supports included university centralised scholarships, financial assistance, tutoring or academic skills support, residential support and counselling services. Some participants reported drawing on these centralised student supports on to specially support Aboriginal and Torres Strait Islander psychology students in their program.

Some participants identified that they were in the planning phase of strategy development, with formalised supports yet to be actioned. In some cases, participants reported that student support strategies were being re-considered or adapted for later release (e.g., “We currently do not have specific strategies in place at present but aim to develop them”). In other instances, participants reported that they did not have any active strategies or support activities available to Aboriginal and Torres Strait Islander psychology students with regards to recruitment, retention and graduation, and/or placement, research, or alumni supports. Limited or no action was most reported in the context of supporting Aboriginal and Torres Strait Islander students transitioning to the workforce, where almost 90% of participants did not have identifiable strategies in place across undergraduate or postgraduate levels.

### Theme 2: Partnership with Aboriginal and Torres Strait Islander peoples, organisations and departments

Participants illustrated the importance of partnerships with Aboriginal and Torres Strait Islander peoples and organisations in facilitating opportunities for students across psychology programs. Participants indicated that the forming and strengthening of partnerships enabled the development of ongoing, innovative and new opportunities for Aboriginal and Torres Strait Islander students. For example, one participant noted in a postgraduate placement setting:

We started, this year, building relationships with Aboriginal and Torres Strait Islander services to create opportunities for MCP [Master of Clinical Psychology] students to undertake placement/s at Aboriginal community-controlled health organisations or related Aboriginal and Torres Strait Islander services. (Participant 16)

Another participant illustrated the importance of partnerships with Aboriginal and Torres Strait Islander organisations and departments across the research setting:

The School has connections with the [Indigenous research organisation] and has engaged in co-supervision of Honours theses with a specific focus on research topics related to health and wellbeing for Aboriginal and Torres Strait Islander peoples. The School has previous hosted Indigenous Cadetships that involve a research component, in collaboration with [IEC at the participant’s university]. (Participant 9)

Partners identified by participants included Aboriginal and Torres Strait Islander psychologists and supervisors, as well as Aboriginal and Torres Strait Islander organisations and departments, in the IEC at the university.

### Theme 3: Research and placement support

In regard to research and placement, at least half of the participants in the study reported they did not have or would like to have support to offer Aboriginal and Torres Strait Islander peoples in these areas. Participants who reported offering research and placement support, identified a range of strategies. For example, participants reported supervision, either by an Aboriginal and Torres Strait Islander person or by a non-Indigenous person. In a placement setting, Participant 15 stated “Indigenous [academic] is also an accredited supervisor and has offered secondary supervision to some placements when there has not been an Aboriginal Clinical Psychologist”. In relation to a search setting, Participant 17 reported “Increasingly, there are non-Indigenous staff who are able to provide a safe space for such research, due to their efforts to build their own competency and sustained interest and willingness to engage in this space”.

Furthermore, participants indicated that placement co-ordinators and program directors provided personalised support to many Aboriginal and Torres Strait Islander students. For example,

The placement co-ordinator will check in regularly with Indigenous students undertaking placement and encourages feedback. If more specialised support may be needed, the placement co-ordinator will liaise with the [Indigenous student support liaison] within our school. (Participant 3)

Finally, many Aboriginal and Torres Strait Islander students were also offered ad hoc support, which was in response to contextual factors and current needs. For example, a participant explained that their “ … department has secured School-level funding to support two students annually to pursue this [placement] training (e.g., lodging, travel)” (Participant 17). Whereas another participant noted:

We have paid for an Aboriginal clinical psychologist to assist by providing cultural consultancy for both the student and the clinical supervisor. This has been used on an ad hoc basis and may not be financially sustainable if numbers increase, but has been welcomed by all involved. (Participant 6)

### Theme 4: Processes supporting cultural safety

Embedding policy and structural supports are often underpinned by deeper values that guide decolonising efforts as they relate to cultural safety. For example, participants reported that they did more than listen to Aboriginal and Torres Strait Islander students, but they also implemented decolonising processes and practices. These decolonising strategies aimed to create a discipline in which Aboriginal and Torres Strait Islander students want to study psychology. Examples provided by participants that coalesced under this theme included embedding culturally responsive marking criteria in honours projects, and the development of Aboriginal and Torres Strait Islander student advisory committees and/or Aboriginal and Torres Strait Islander students’ contributing to School committees. Participant 5 noted, “We are currently pursuing changes to the honours marking criteria to ensure it is clear that [Indigenous research projects] of this nature are not only permissible, but encouraged”.

Participant 6 noted, “A student reference group – this is to have input into the curriculum, but also serves the purpose of showing we are listening to students and also aims to make those students feel included and provide a culturally safe environment”.

Participant 17 noted, “Our Departmental culture highly values student voice, which has created opportunities for students to actively share their ideas, perspectives and concerns. This has influenced Department activities (e.g., Indigenous students led a reflective session on cultural humility in higher education teaching during NAIDOC week for staff). Other systemic examples were provided by participants, which included engaging staff in cultural responsiveness training and increasing Indigenous representation across curricula and staff, with the aim of building the capacity of staff to support Aboriginal and Torres Strait Islander students. For example, Participant 5 reported, “Cultural competence training for staff appears to have led to some important changes in students finding psychology to be a culturally safe space. The addition of a core course on culture in the undergraduate curriculum has also contributed to this”.

Participant 16 reported, “Creating a safe environment & validating Indigenous ways of being-knowing-doing through decolonising & Indigenising the curriculum in terms of content and pedagogies – & points to the importance of building the capacity of psychology academics to decolonise and Indigenise”.

## Discussion

Aboriginal and Torres Strait Islander psychology students lived experiences are diverse. Strategies and initiatives that accommodate this diversity should be implemented to support recruitment, retention and graduation. The current research examined strategies and actions undertaken by psychology HEPs to support Aboriginal and Torres Strait Islander psychology students. This study included all levels of psychology study, from undergraduate to honours, and postgraduate study, and nuanced areas of support, including research and placement supports. A range of activities and actions to support Aboriginal and Torres Strait Islander students are underway and reflected in the themes. The findings coalesced into the following four key themes: policies and structural support; partnerships with Aboriginal and Torres Strait Islander peoples, organisations and departments; research and placement support; and processes supporting cultural safety. Whilst work is underway, it is important to note that a proportion of participants reported that work was yet to be undertaken at the time of the survey. However, the identification of these strategies will strengthen the implementation of systemic changes, both in policy and practice.

The findings in this study were consistent with the literature and other research in allied health, such as nursing (West et al., 2016) and medicine (Garvey et al., 2009), and across higher education contexts, including when examining the development of pathway opportunities (Andrews et al., 2023; Ohan, McMullen et al., 2023), understanding the factors involved in student retention (Taylor et al., 2019) and reviewing the success factors for completion of higher education (Fredericks et al., 2022; Milne et al., 2016). Collectively, research in these areas were unified in their messaging: multipronged, holistic and layered support and strategies are required to effectively support Aboriginal and Torres Strait Islander students in their educational journeys. This may include a broad range of initiatives such as financial aid, academic support, recruitment initiatives, partnerships with Indigenous organisations, social support and systemic level transformations that actively work to eliminate racism and facilitate cultural safety across all levels of the university (Fredericks et al., 2022; Taylor et al., 2019).

One unique finding of the present findings was the role of equity pathways as part of the structural supports in enabling more Aboriginal and Torres Strait Islander students to progress in their psychology studies. The current pathway to psychology registration requires an honours or equivalent accredited 4-year psychology sequence, followed by postgraduate training (higher degree pathway or 5 + 1 internship pathway; Psychology Board of Australia, 2024b). Additionally, training to become a psychologist with an area of practice speciality requires completion of an accredited postgraduate degree approved for area of practice endorsement (Psychology Board of Australia, 2024c). As explained by Ohan, McMullen et al. (2023), both of these entry points, especially in masters programs, are highly competitive and challenging as a result of the resource-intensive nature of the programs. Furthermore, there is a bottleneck of participation, where Aboriginal and Torres Strait Islander representation in honours and postgraduate studies substantially taper. Increasing Aboriginal and Torres Strait Islander students’ accessibility and progression to these psychology programs is, therefore, extremely important. The current study’s finding that equity pathways were actively being employed to support students highlights the nuances of psychology training in Australia and consequently, the necessity of equity pathways in supporting some Aboriginal and Torres Strait Islander students. Empowering more Aboriginal and Torres Strait Islander students to progress in their studies requires targeted supports across psychology placement and research areas. This is especially relevant across postgraduate studies, where these areas form a substantial component of course requirements in psychology. These findings, along with other work conducted in AIPEP (see Selkirk et al., 2024), provide illustrations and practical strategies for psychology HEPs.

An interesting finding in this research was the absence of alumni supports for Aboriginal and Torres Strait Islander students transitioning to the workforce. Supporting Aboriginal and Torres Strait Islander students to successfully transition to the workforce is an important step in the student journey. This is particularly helpful in ensuring Aboriginal and Torres Strait Islander students have the knowledge of the types and breadth of opportunities that exist beyond their psychology degree, and ensuring that students feel ready to navigate the new challenges that the workplace brings. Together, this finding highlighted the need for greater focus on workforce supports post-graduation, across both the undergraduate and postgraduate levels. More research is required to explore these issues, including support post-graduation.

### Limitations and future research

The current study provides a snapshot of the strategies and initiatives undertaken by 18 of 41 psychology HEPs in supporting Aboriginal and Torres Strait Islander psychology students. Participants who completed the survey were a representative of their HEP and were not asked to identify if they were an Aboriginal and/or Torres Strait Islander person. Given the potential that many participants were likely non-Indigenous, responses provided in relation to Indigenous knowledges, decolonising and cultural safety must be considered in that context. Future research could explicitly engage the voices of Aboriginal and Torres Strait Islander stakeholders who contribute to psychology higher education, for example students, staff, IEC and more. Further research also could seek to capture the initiatives and experiences of the 23 HEPs that did not participate in the current study.

While the current study touched some of the underlying barriers experienced by some Aboriginal and Torres Strait Islander psychology students, a more comprehensive analysis of the psychology-discipline specific barriers would be beneficial. Taking a strength-based perspective in exploring and addressing discipline specific barriers is important. The current study also did not directly explore the specific social and emotional wellbeing factors pertaining to Aboriginal and Torres Strait Islander psychology students, which is likely to be a key element in student experience in their psychology education. To further explore factors contributing to students’ continuation in psychology, possible future research could include a comprehensive exploration of Aboriginal and Torres Strait Islander psychology students SEWB and lived experiences in their psychology education. Engaging Aboriginal and Torres Strait Islander psychology student voices is an important piece in cultivating cultural safety within HEPs. Though this should be done in tandem with the multipronged strategies as outlined in this study, such as engaging with Indigenous leadership and creating opportunities for Indigenous academics within the school of psychology.

## Conclusion

Psychology HEPs play a critical role in increasing Aboriginal and Torres Strait Islander representation and participation in the discipline psychology and psychology workforce. Many Aboriginal and Torres Strait Islander psychology students experience systemic, cultural, political and economic barriers that other student cohorts do not have to navigate during their studies. Therefore, psychology HEPs (individually and collectively) have a responsibility to increase the enablers and reduce the barriers for many Aboriginal and Torres Strait Islander students. Previous research, combined with findings from the current study, collectively indicate that psychology HEPs need to engage in multipronged, holistic and innovative strategies to effectively support the diverse needs of Aboriginal and Torres Strait Islander psychology students in their educational journeys from entry to graduation. It is evident from this study that HEPs are actively working to improve the experiences of many Aboriginal and Torres Strait Islander psychology students, and this research study will facilitate that work. Though there is a way to go, and the diverse needs of Aboriginal and Torres Strait Islander psychology students are likely to evolve over time. It is recognised that each psychology HEP will have its relative strengths and areas for growth in supporting Aboriginal and Torres Strait Islander psychology students. Further, multipronged and multilayered initiatives take time, resourcing and collaboration to develop and implement successfully.

The findings from the current research provide a snapshot of initiatives that are being implemented across Australia to support Aboriginal and Torres Strait Islander psychology students. This provides a useful point of reference for HEP’s to critically examine their current initiatives and whether improvements can be made to support Aboriginal and Torres Strait Islander psychology students within their place-based context. The hope is that information will provide ideas and guidance regarding how HEPs can support Aboriginal and Torres Strait Islander psychology students, in turn increasing the representation of Aboriginal and Torres Strait Islander psychology peoples in the discipline and contributing to a more robust and culturally safe psychology workforce.

## Supplementary Material

Supplemental Material

## Data Availability

In line with the Ethics approval for this project, data are not publicly available for this project.

## Authors’positionalities

B.S. is a Noongar woman living and working on Wadandi-Noongar Boodjar, an experienced clinical psychologist with a strong focus on culturally informed care and decolonising practices, and is a research fellow with the School for Indigenous Studies at the University of Western Australia focusing on Indigenous psychology and social and emotional wellbeing. P.D is from Bardi people of the Kimberley. P.D. specialises in Indigenous psychology, mental health, and education and is recognised as being among the leading world experts on social and emotional wellbeing and suicide prevention. P.D. is a professor in the School of Indigenous Studies, chief investigator of the Transforming Indigenous Mental Health and Wellbeing research project, and the director of the national Centre of Best Practice in Aboriginal and Torres Strait Islander Suicide Prevention at the Poche Centre for Indigenous Health at the University of Western Australia. C.G is a Gamilaraay woman who is a research fellow with the School of Indigenous Studies at the University of Western Australia. C.G. has over 20 years of experience working with Aboriginal and Torres Strait Islander social and emotional wellbeing, with expertise in working with older people and supporting culturally responsive service delivery. C.G. is an occupational therapist and has led initiatives in her profession to support the inclusion of Indigenous health curricula in occupational therapy programs. J.A. has Cypriot heritage, was born and raised in Larrakia Country, and now living and working on Whadjuk Noongar Boodja. J.A. is a research fellow with the School for Indigenous Studies at the University of Western Australia, where she has been working with Aboriginal and Torres Strait Islander peoples in research over the past three years, with an emphasis on social and emotional wellbeing, education, and decolonising praxis.

Note: The authorship order was decided by and led by the Indigenous leadership and governance in the project, and in consultation with the authors.
