# Supplementary material for: Decolonising tertiary psychology student support in Australia: empowering Aboriginal and Torres Strait Islander psychology students
Source: Aust J Psychol. 2025 Mar 24;77(1):2478083. doi: 10.1080/00049530.2025.2478083 (PMC12218500; doi:10.1080/00049530.2025.2478083)
Supplement: Supplemental Material [file RAUP_A_2478083_SM7122.docx]

**Supplementary Material**

Table 1: Strategies and actions being undertaken by higher education providers to support Aboriginal and Torres Strait Islander psychology students.

| Theme & Description | Example |
| --- | --- |
| *Theme 1: Policies and Structural Support*  Policies, procedures, and integrated structural support that facilitate the recruitment, retention, and graduation of Aboriginal and Torres Strait Islander psychology students.^[[1]](#footnote-1)^ | *Schools of psychology*   - Admission policies - Reserved places in psychology programs - Pathways - Scholarships - Mentoring programs   *University-wide initiatives*   - University centralised scholarships - Financial aid - Website resources - Tutoring or academic skills support - Residential support - Counselling services |
| *Theme 2: Partnership with Aboriginal and Torres Strait Islander Peoples, Organisations, and Departments*  Relationship building and partnerships with Aboriginal and Torres Strait Islander peoples, communities, organisations, and departments, including the Indigenous Education Centre at the university.  Collaborative partnerships facilitate ongoing, innovative, and new opportunities/supports for Aboriginal and Torres Strait Islander students undertaking research and placements in psychology, across undergraduate, Masters, and PhD levels. | - Collaborative research cadetship - Joint research supervisory arrangements and opportunities between Indigenous departments and higher education providers - Placement opportunities at Aboriginal and Torres Strait Islander health services |
| *Theme 3: Research and Placement Support*  Supports and provisions for Aboriginal and Torres Strait Islander psychology students undertaking research and placement opportunities. This relates to support led and delivered by the higher education provider. | - Supervision by Aboriginal and Torres Strait Islander psychologists and educators, and non-Indigenous staff with learning in cultural responsiveness - Dedicated placement co-ordinator - Financial support and housing provisions on rural placements - Ad-hoc support, such as student check ins, arrangements for specialist and cultural consultancy support |
| *Theme 4: Processes Supporting Cultural Safety*  Broader systems approach and ethos in advocating for students, listening to the student voice about what they need, and broadly working towards decolonising psychology to create a discipline where Aboriginal and Torres Strait Islander students want to study psychology. | - Listening to Aboriginal and Torres Strait Islander students’ needs and aspirations for student support - Indigenous representation procedures - Culturally responsive marking criteria - Hiring and increasing representation of Aboriginal and Torres Strait Islander staff - All staff capacity building - Embedding decolonising theory and practice in curricula and ways of working with students |

1. Some participants noted engaging in planning regarding strategies to support Aboriginal and Torres Strait Islander psychology students, with formalised supports under development and/or yet to be implemented. [↑](#footnote-ref-1)
